# Electroacupuncture-induced reduction of myocardial ischemia–reperfusion injury via FTO-dependent m6A methylation modulation

**DOI:** 10.1515/med-2025-1255

**Published:** 2025-08-19

**Authors:** Ying-Hua Zou, En Zhou, Chen-Long Xie, Long Sun, Yu-Jiao Zhang, Chun-Chun Wang, Xing Li, Jun Guo

**Affiliations:** Department of Anesthesiology, Shuguang Hospital Affiliated to Shanghai University of Traditional Chinese Medicine, Shanghai, 201203, China; Department of Cardiovascular Surgery, Shanghai Ninth People’s Hospital, School of Medicine, Shanghai Jiao Tong University, Shanghai, 200000, China; Department of Anesthesiology and Intensive Care Medicine, Xinhua Hospital, School of Medicine, Shanghai Jiao Tong University, Shanghai, 200000, China; Department of Anesthesiology, Shuguang Hospital Affiliated to Shanghai University of Traditional Chinese Medicine, 528 Zhangheng Road, Pudong New Area, Shanghai, 201203, China

**Keywords:** cell apoptosis, electroacupuncture stimulation, FTO, m6A, myocardial ischemia–reperfusion injury

## Abstract

**Objective:**

This study aims to investigate the potential of electroacupuncture to mitigate myocardial ischemia–reperfusion injury (MIRI) by influencing N6-methyladenosine (m6A) methylation through modulation of the fat mass and obesity-associated protein (FTO).

**Methods:**

An experimental murine model of MIRI was established by surgically occluding the left anterior descending coronary artery, followed by reperfusion. Electroacupuncture treatment targeting Neiguan acupoints was administered 7 days before ischemia induction. Cardiac function was evaluated using echocardiography, and myocardial infarction size was assessed through Evans Blue and triphenyltetrazolium chloride dual staining. To measure m6A methylation and mRNA expression of FTO and mediator complex subunit 1 (Med1), RNA immunoprecipitation and quantitative polymerase chain reaction were utilized. Western blot analysis was conducted to determine the protein expression levels of Med1, Bcl-2, and Bax.

**Results:**

Electroacupuncture pretreatment was associated with a reduction in myocardial injury, demonstrated by preserved ejection fraction and reduced infarct size. Enhanced FTO expression and decreased m6A methylation were observed in myocardial tissue following electroacupuncture treatment. Additionally, Med1 – a downstream target of m6A – exhibited decreased mRNA expression in the electroacupuncture-treated group, correlating with reduced cardiomyocyte apoptosis.

**Conclusion:**

Electroacupuncture pretreatment may confer cardioprotective effects in MIRI by upregulating FTO, thereby modulating m6A methylation and reducing Med1 expression.

## 1 Introduction

Myocardial ischemia–reperfusion injury (MIRI) is characterized by significant structural and functional deterioration in myocardial tissue within the ischemic region, arising upon reperfusion following prolonged interruption of blood supply [1]. MIRI frequently occurs in clinical settings, including coronary artery bypass graft surgery, various direct cardiac procedures, percutaneous coronary interventions, thrombolysis, and instances of prolonged intraoperative hypotension. Notably, previous studies indicate that an intraoperative mean arterial pressure below 60 mmHg, sustained for over 30 min, correlates with incidence rates of postoperative myocardial injury, myocardial infarction, and 30-day mortality at 29, 6, and 4%, respectively [2]. Thus, perioperative myocardial injury from ischemia–reperfusion represents a significant factor in poor prognoses and heightened mortality rates.

Despite the clinical relevance of MIRI, existing therapeutic approaches remain limited. Techniques such as ischemic preconditioning (IPC) and ischemic postconditioning are available but have notable limitations. IPC, though effective in preventing MIRI, is challenging to apply more widely due to the requirement for direct cardiac intervention and the risk of thromboembolic events from clamping an atherosclerotic aorta [3]. Consequently, advancing research into novel therapeutic strategies and expanding the conceptual understanding of MIRI management is essential.

Acupuncture, rooted in traditional Chinese medicine, has demonstrated beneficial cardiovascular effects through a neurohumoral mechanism known as the long-loop pathway [4,5]. Clinical studies have highlighted that acupuncture may improve quality of life for individuals with heart failure [6]. With advancements in medical technology, electroacupuncture has evolved into an established treatment modality. Preoperative electroacupuncture has been shown to reduce the release of cardiac troponin I in both adult and pediatric cardiac surgery patients, while also decreasing their duration of stay in the intensive care unit [7,8]. Previous findings from our research further support that electroacupuncture attenuates pro-inflammatory responses and myocardial injury during the reperfusion phase [9].

Despite progress in this area, understanding of the precise mechanisms underpinning the cardioprotective effects of electroacupuncture against MIRI remains incomplete. Recent studies have identified N6-methyladenosine (m6A) RNA modification as a critical factor in RNA splicing, export, stability, and translation, marking it as the most prevalent epigenetic methylation modification in mRNAs and non-coding RNAs [10,11].

Emerging evidence indicates that m6A modification plays a significant role in cardiovascular disease processes, including the regulation of myocardial ischemia/reperfusion (I/R) injury, cardiac remodeling, and cardiomyocyte contractile function [12–14]. The dynamic and reversible nature of m6A modification underscores its regulatory importance across cellular functions.

The regulation of m6A modification in RNA involves three main categories of regulatory proteins: “writers” (methyltransferases), which introduce the m6A mark; “erasers” (demethylases), which remove it; and “readers” (m6A binding proteins), which detect and interpret the presence of m6A [15,16]. The demethylase activity of m6A “erasers” requires iron as a cofactor and α-ketoglutaric acid as a co-substrate to catalyze the removal of m6A modifications from RNA [17]. Under physiological homeostasis, the function of these m6A “erasers” appears to be tightly regulated and limited in scope [15].

Currently, fat mass and obesity-associated protein (FTO) and α-ketoglutarate-dependent dioxygenase AlkB homologue 5 are the only identified enzymes known to catalyze m6A demethylation in RNA. *In vitro* studies demonstrate that overexpression of FTO has a protective effect, mitigating hypoxia/reoxygenation (H/R)-induced cardiomyocyte apoptosis and inflammation, thereby contributing to MIRI attenuation [18]. While the specific pathways through which FTO modulates MIRI are not fully understood, ongoing research seeks to clarify the therapeutic potential of electroacupuncture as a modulator of m6A levels through FTO activation. This study aims to investigate whether electroacupuncture influences m6A expression via FTO, potentially ameliorating MIRI.

## 2 Methods

### 2.1 Ethical consideration

All animal procedures adhered to protocols approved by the Committee on the Ethics of Animal Experiments at Shuguang Hospital, affiliated with Shanghai University of Traditional Chinese Medicine. Surgical interventions and euthanasia were conducted under anesthesia, utilizing bilateral thoracotomy to ensure humane treatment. Mice were randomly divided into sham, I/R, and I/R + EA groups of five mice each.

### 2.2 Electroacupuncture pretreatment

Electroacupuncture treatment commenced 7 days prior to ischemia induction, with stimulation applied to the Neiguan acupoints (PC6) located on the ventral aspect between the radius and ulna, approximately 1 mm proximal to the forelimb wrists (Figure S1). Conscious mice were securely positioned on an experimental platform, and bilateral acupuncture was administered subcutaneously at a depth of approximately 2 mm at the PC6 points. Limb tremors were monitored to confirm effective stimulation. Electroacupuncture was performed using a continuous wave setting, with parameters of 2 Hz frequency and 0.5 mA intensity, sustained for 30 min. Electroacupuncture treatment was administered once daily for 7 consecutive days before ischemia induction. Each session was applied for 30 min, and the final electroacupuncture treatment was conducted 30 min before the surgical procedure. Control group mice received the procedure at non-meridian points located 1 mm proximal to the elbow along the dorsal midline, in a region devoid of significant acupoints. Within 30 min of electroacupuncture administration, surgical ligation of the left anterior descending artery was initiated to maximize treatment efficacy (Figure S2).

### 2.3 *In vivo* MIRI model

To induce MIRI, mice were anesthetized with 5% isoflurane and intubated to maintain continuous anesthesia via mechanical ventilation. Myocardial ischemia was induced by ligation of the LAD artery with a 7-0 silk suture, followed by reperfusion after a 30 min ischemic interval achieved by releasing the ligature.

### 2.4 Cardiac imaging and echocardiographic assessment

Echocardiographic assessments were performed on 3 days following reperfusion, using the Vevo 770 high-resolution imaging system. These assessments enabled measurement of left ventricular wall thicknesses and chamber dimensions during both systole and diastole. Ejection fractions (EFs) were calculated automatically based on these measurements.

### 2.5 Evaluation of infarct size using staining techniques

Infarct delineation was achieved by occluding the left anterior descending artery at the original ligation site 3 days post-reperfusion, followed by infusion of 1% Evans Blue dye through the abdominal aorta. The left ventricle was subsequently excised, rinsed, and sectioned into evenly sized slices. Each slice was incubated in a 1% solution of 2,3,5-triphenyltetrazolium chloride (TTC) at 37°C for 15 min, then fixed in formalin. Infarcted and at-risk myocardial areas were quantified through imaging analysis, adjusted by slice weight to determine the extent of myocardial involvement. Ratios of the area at risk to total left ventricular area and infarct size to area at risk were calculated and expressed as percentages for subsequent statistical analysis.

### 2.6 *In vitro* H/R model

To simulate H/R conditions, cardiomyocytes were subjected to a 12 h hypoxic environment (1% O_2_, 5% CO_2_, 94% N_2_) followed by a 6 h reoxygenation period (5% CO_2_, 21% O_2_, 74% N_2_).

### 2.7 RNA immunoprecipitation and quantitative PCR analysis (MeRIP-qPCR)

Cardiomyocytes were lysed, and total RNA was extracted using Trizol reagent. A total of 100 μg of RNA was incubated with either anti-m6A antibody or control IgG on protein A/G beads in MeRIP buffer. m6A-enriched RNA was isolated and subsequently quantified using quantitative reverse transcription PCR (qRT-PCR).

### 2.8 Quantitative PCR technique

Following RNA extraction via Trizol reagent, the RNA was reverse transcribed into complementary DNA using a thermal cycler. Quantification of target mRNA was conducted through real-time PCR with Takara reverse transcription kits and SYBR Green II, using specific primers provided by Sangon Biotech. mRNA expression levels were assessed using the 2^–ΔΔCt^ method.

### 2.9 m6A modification quantification

Total RNA integrity was verified with the NanoDrop system, and global m6A mRNA levels were quantified using the EpiQuik m6A Methylation Quantification Kit. This assay involved a colorimetric measurement of m6A enrichment at an absorbance of 450 nm.

### 2.10 RNA immunoprecipitation-PCR (RIP-qPCR)

Cardiomyocytes were lysed in RIP lysis buffer, and m6A-binding RNAs were immunoprecipitated using the EZ Magna RIP Kit with specific antibodies. The enriched RNAs were analyzed using qRT-PCR and normalized to input controls for quantification.

### 2.11 Western blot analysis for protein quantification

Proteins were extracted from cardiac tissues using RIPA buffer, separated by SDS-PAGE, and transferred onto polyvinylidene difluoride membranes. Membranes were incubated with primary antibodies targeting FTO, mediator complex subunit 1 (Med1), Bcl-2, Bax, and GAPDH. Detection involved horseradish peroxidase-conjugated secondary antibodies, and visualization was performed using an enhanced chemiluminescence detection system.

### 2.12 Statistical analysis

Quantitative data are presented as mean ± standard error of the mean. Comparative analyses were performed using analysis of variance, followed by least significant difference *post-hoc* tests via SPSS software, version 13.0. A *P*-value of less than 0.05 was considered statistically significant.


**Ethical approval:** The protocol for all animal procedures received approval from the Committee on the Ethics of Animal Experiments at Shuguang Hospital, affiliated with Shanghai University of Traditional Chinese Medicine, and were conducted in compliance with the Animal Research: Reporting of *In Vivo* Experiments (ARRIVE) guidelines (Approval number: 2022-989-62-01).

## 3 Results

### 3.1 Attenuation of MIRI via electroacupuncture-induced modulation

**Figure 1:**
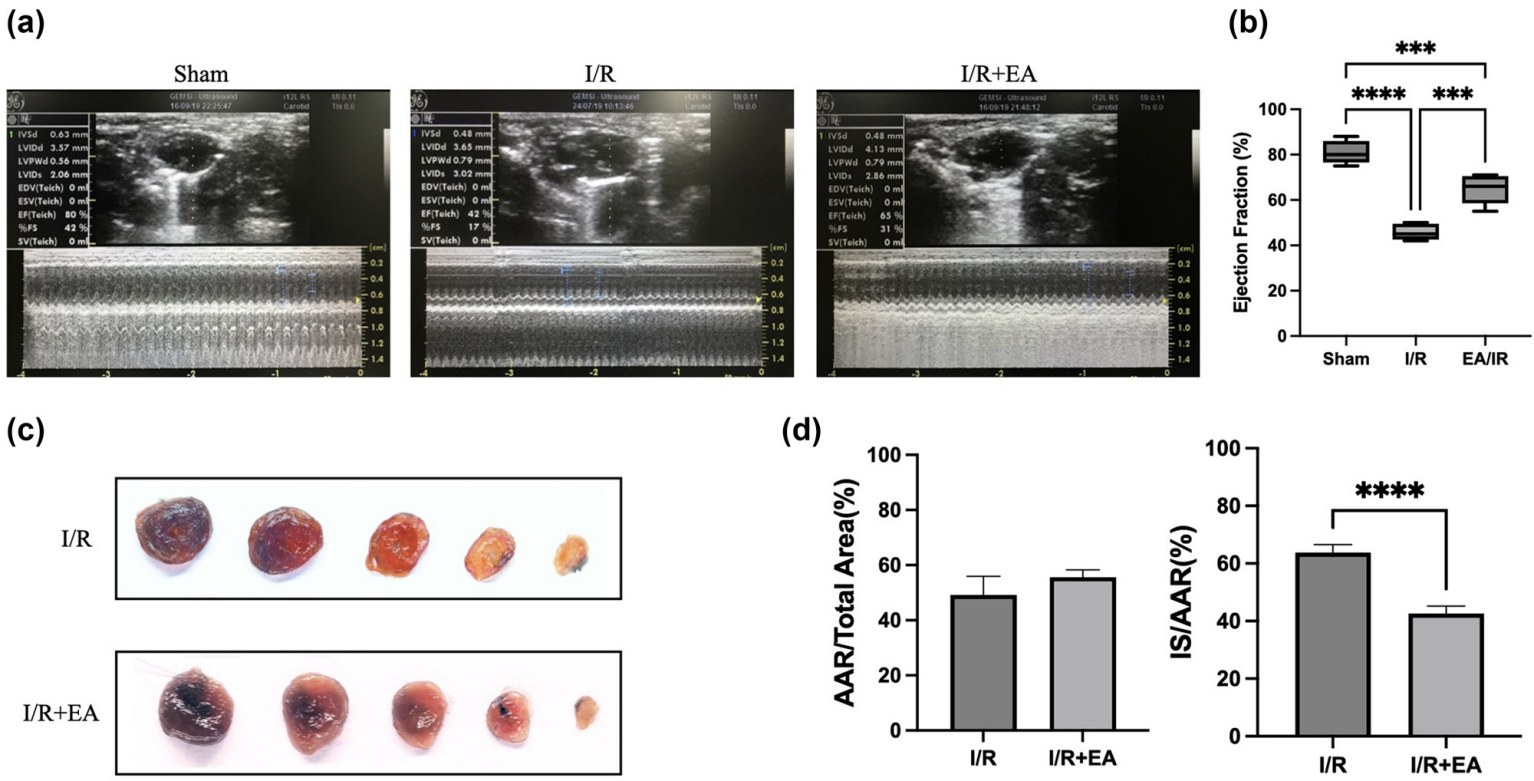
Electroacupuncture attenuates the decline in cardiac function and reduces MIRI area. (a) and (b) Echocardiographic assessment of cardiac function; (c) and (d) Evans Blue/TTC staining of myocardial tissue in mice (Sham: Control group; I/R: Mice with MIRI; I/R + EA: mice with MIRI following electroacupuncture treatment) (*n* = 5, *P < 0.05, **P < 0.01, ***P < 0.001, ****P < 0.0001).

Mice subjected to I/R injury displayed a significant reduction in EF compared to the sham group. However, this reduction was attenuated in mice pretreated with electroacupuncture, as shown in Figure 1a and b. Evans Blue/TTC staining revealed a marked reduction in the infarct size relative to the area at risk in the myocardium of mice in the I/R + EA group compared to the I/R injury group, as illustrated in Figure 1c and d.

### 3.2 Electroacupuncture stimulation enhances cardiac FTO levels

**Figure 2:**
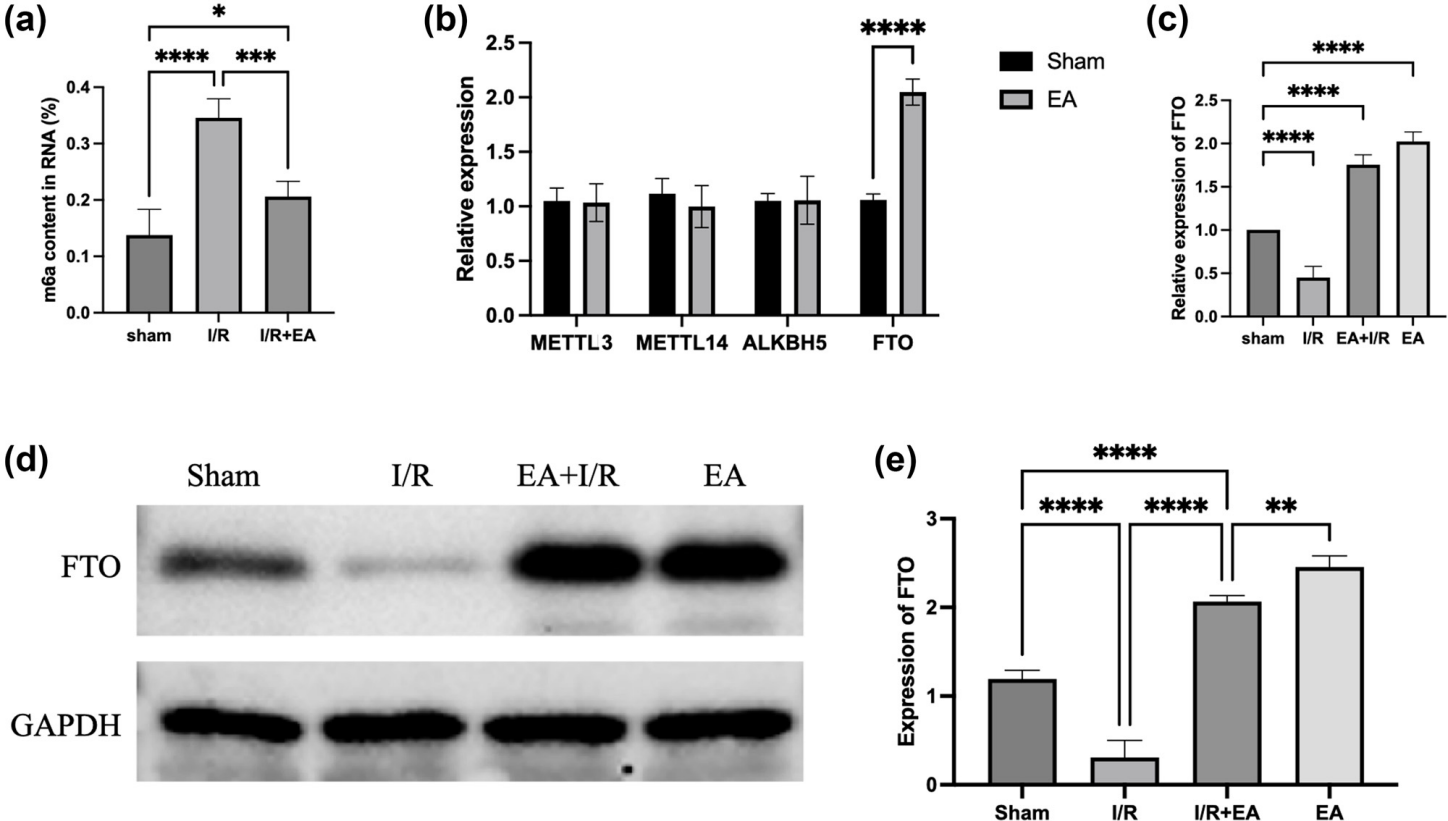
Electroacupuncture stimulation enhances FTO expression in mouse myocardial tissue. (a) Total RNA m6A content in myocardial tissue of I/R, I/R + EA, and control mice. (b) Expression of m6A modification-related proteins in myocardial tissue. (c) RT-PCR results showing FTO expression in myocardial tissue. (d) and (e) Western blot analysis of FTO expression in myocardial tissue (Sham: Control group; I/R: Mice with MIRI; I/R + EA: Mice with MIRI following electroacupuncture treatment; EA: mice receiving only electroacupuncture) (*n* = 5, *P < 0.05, **P < 0.01, ***P < 0.001, ****P < 0.0001).

In myocardial tissues of mice exposed to I/R injury, levels of m6A modification were notably elevated. In contrast, electroacupuncture-pretreated mice did not exhibit a substantial increase in m6A expression following I/R injury, as shown in Figure 2a. Electroacupuncture intervention was also associated with a marked upregulation of FTO expression in cardiac tissue, as illustrated in Figure 2b.

Furthermore, in the I/R injury group, both FTO mRNA and protein levels were decreased. However, electroacupuncture treatment significantly elevated both FTO mRNA and protein levels, even in the presence of prior I/R injury, as displayed in Figure 2c–e.

### 3.3 Modulatory effects of electroacupuncture on m6A modification and targeting of Med1

**Figure 3:**
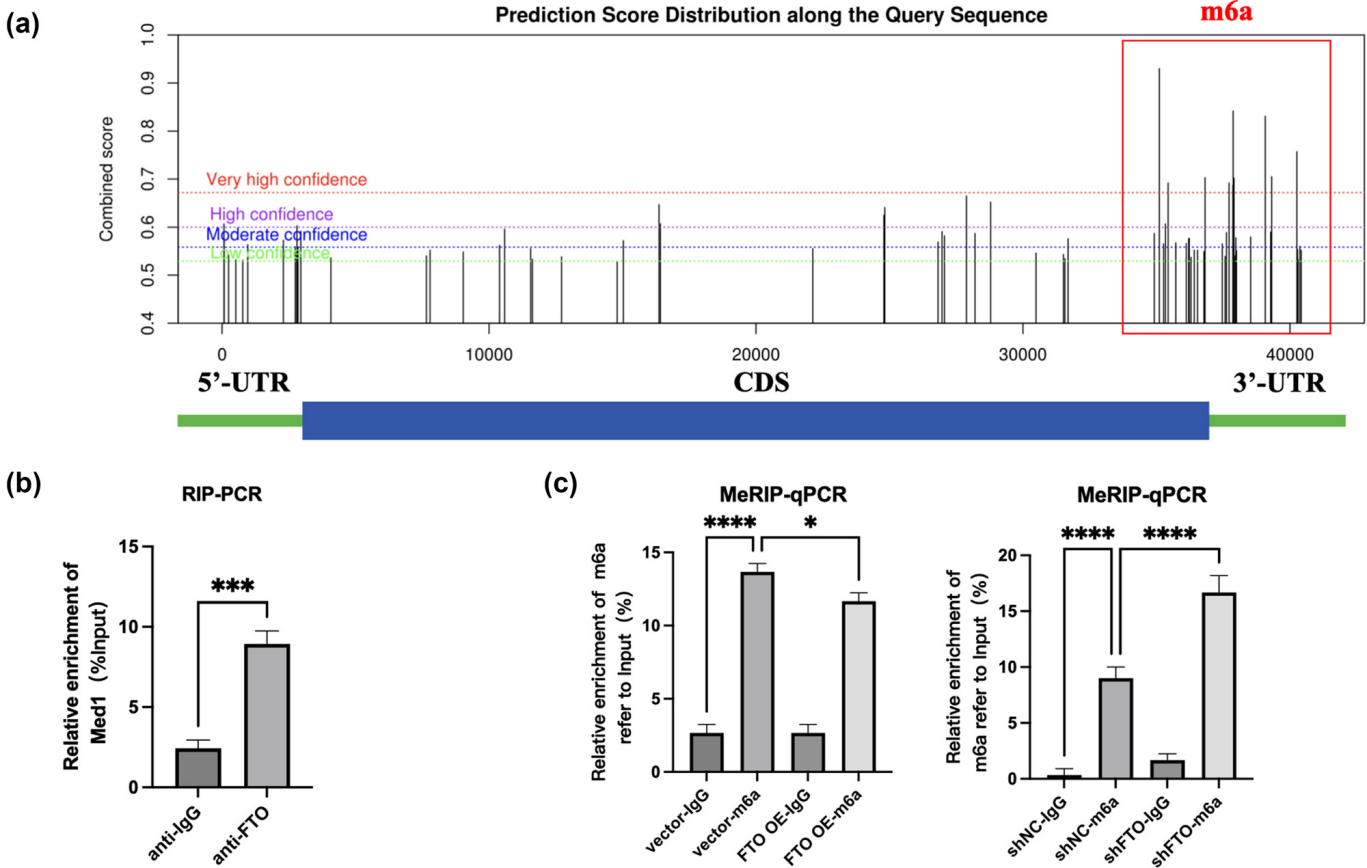
Electroacupuncture stimulation reduces m6A expression, with Med1 identified as a potential target. (a) SRAMP predictive analysis (http://www.cuilab.cn/sramp) indicating high-probability m6A modification sites in the 3′-UTR of Med1. (b) RIP-PCR analysis showing increased Med1 mRNA enrichment when isolated with anti-FTO antibody. (c) MeRIP-PCR assay indicating m6A modification levels in cardiomyocytes subjected to H/R, dependent on FTO overexpression or reduction (*n* = 5, *P < 0.05, **P < 0.01, ***P < 0.001, ****P < 0.0001).

Analysis using the SRAMP online predictive platform (http://www.cuilab.cn/sramp) identified multiple high-probability m6A sites within the 3′-UTR of Med1 (Figure 3a). Immunoprecipitation with the FTO antibody enriched Med1 mRNA, confirming interaction as shown in Figure 3b. Overexpression of FTO correlated with reduced m6A modification levels, while FTO suppression led to increased m6A levels in cardiomyocytes subjected to H/R stress, as depicted in Figure 3c.

### 3.4 Electroacupuncture-induced regulation of Med1 methylation influences hypoxia–reoxygenation-associated cellular mortality

**Figure 4:**
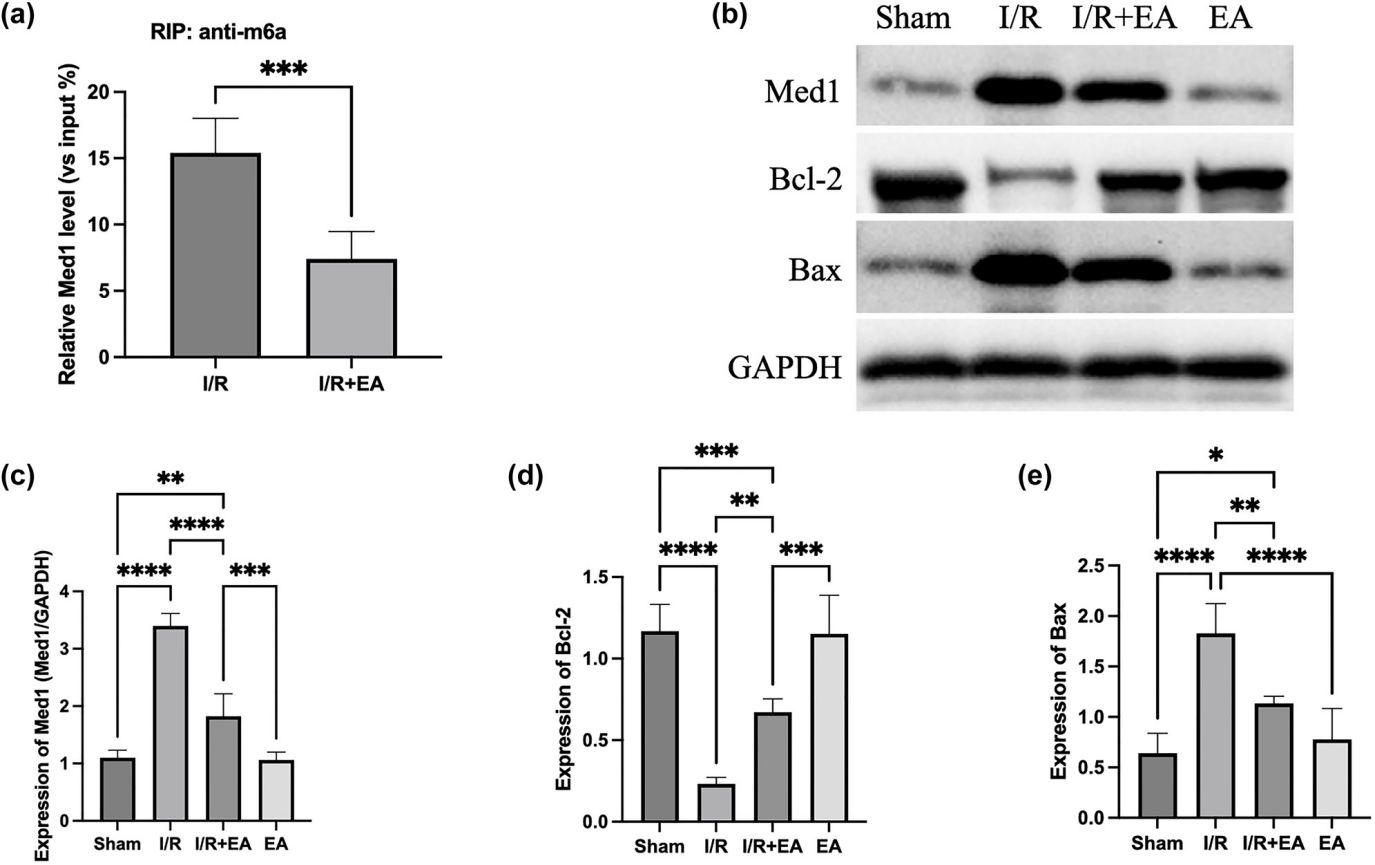
Effects of electroacupuncture on m6A modification of Med1 and expression of apoptosis-related proteins in myocardial tissue following I/R injury. (a) RNA immunoprecipitation assay showing relative m6A modification levels of Med1 in the I/R and I/R+EA groups. (b–e) Western blot analysis of Med1, Bcl-2, and Bax expression in mouse myocardial tissue (Sham: Control group; I/R: Mice with MIRI; I/R + EA: Mice with MIRI following electroacupuncture treatment; EA: Mice receiving only electroacupuncture) (*n* = 5, *P < 0.05, **P < 0.01, ***P < 0.001, ****P < 0.0001).

Previous studies have demonstrated that Med1 m6A modification is elevated in MIRI [19]. In contrast, our results show that electroacupuncture (EA) pre-treatment significantly reduced Med1 methylation levels (Figure 4a), suggesting a potential protective role of EA in alleviating MIRI through the regulation of Med1 methylation. Med1 expression was significantly elevated in MIRI, in agreement with findings from prior studies. In myocardial specimens from I/R-injured mice that received electroacupuncture pretreatment, there was a substantial increase in Bcl-2 expression along with a reduction in Bax expression, as depicted in Figure 4.

## 4 Discussion

Myocardial infarction is a leading cause of global mortality in cardiovascular diseases, with timely reperfusion therapy remaining the primary intervention for restoring blood flow to obstructed vessels. Despite its efficacy in revascularization, reperfusion may induce MIRI, which can lead to multiple adverse outcomes that negatively influence patient prognosis [3]. Presently, effective preventive strategies for MIRI are limited.

Electroacupuncture, an accessible and non-invasive therapeutic modality rooted in traditional Chinese medicine, has garnered substantial empirical support for efficacy across various clinical conditions [20–22]. Numerous studies have demonstrated that electroacupuncture can alleviate ischemia–reperfusion injury in organs such as the heart and brain [9,23,24]. Specifically, electroacupuncture has been shown to reduce MIRI by inhibiting mitophagy and suppressing pro-inflammatory responses [9,25]. In this study, electroacupuncture pretreatment significantly reduced MIRI-related myocardial injury in mice, as evidenced by a reduction in infarct size and preservation of EF following I/R injury. These findings align with previous research, supporting the cardioprotective role of electroacupuncture in MIRI models [9].

Recent research has underscored the broad influence of m6A modification in various pathophysiological processes, including myocardial infarction and myocardial fibrosis. This post-transcriptional modification plays a significant role in RNA splicing, stability, and cell differentiation. FTO, a key enzyme in RNA demethylation, has emerged as a critical regulator across a range of pathological and physiological events [18]. Notably, recent studies reveal that moxibustion – a traditional Chinese therapeutic technique – can modulate m6A methylation in murine brain models, thereby enhancing cognitive and memory functions [26].

Previous neurological studies have shown that electroacupuncture may regulate m6A modifications to confer protection in cerebral ischemia–reperfusion injury [27]. In alignment with these findings, this study demonstrates that electroacupuncture pretreatment significantly increases FTO expression in myocardial tissue. Prior research has established that FTO expression is elevated in myocardial infarction models, where it contributes to cardiac homeostasis, structural remodeling, and reparative processes [13]. Moreover, existing evidence suggests that FTO mitigates MIRI through mechanisms mediated by m6A modification [18]. However, direct therapeutic methods to precisely regulate m6A levels in clinical settings remain a challenge.

Our investigation revealed a significant increase in m6A methylation levels within the cardiac tissues of mice subjected to I/R injury. Notably, electroacupuncture administered prior to the induction of I/R effectively counteracted this elevation in m6A levels. These findings suggest that electroacupuncture may modulate m6A abundance in myocardial cells via regulation of FTO expression, thereby attenuating the impact of MIRI in murine models.

The results of this study indicate that electroacupuncture therapy provides an efficient, straightforward, and safe approach for modulating m6A methylation levels within cardiac cells. Additionally, findings suggest that Med1 may act as a downstream effector of m6A modifications, influencing MIRI pathogenesis. Previous studies have demonstrated that suppressing Med1 expression significantly reduces MIRI severity in murine models [19].

Med1, also known as TRAP220 in murine models and RB18A in human studies, is essential for murine survival, largely due to its regulatory role over genes involved in cardiac metabolism and mitochondrial function [28,29]. Cell apoptosis, a key process in MIRI, is primarily regulated through two main pathways: the extrinsic (death receptor-mediated) and intrinsic (mitochondrial) pathways [30–32]. The regulation of mitochondrial apoptotic events is largely governed by Bcl-2 family proteins, which include anti-apoptotic members like Bcl-2 and pro-apoptotic members like Bax. These proteins control cytochrome c release from mitochondria, primarily by modulating mitochondrial membrane permeability [31,32]. In this study, we explored the regulatory effects of electroacupuncture on Med1, a key component of the mediator complex, known to play a critical role in cardiac function and apoptosis regulation. Our findings suggest that electroacupuncture may suppress Med1 expression, thereby mitigating myocardial apoptosis during ischemia–reperfusion injury. This suppression is likely mediated through m6A methylation mechanisms, with FTO acting as an essential regulator. FTO, an m6A demethylase, can demethylate mRNA, leading to the downregulation of Med1, which is encoded by TRAP220 in the myocardium. This reduction in Med1 expression could interfere with the apoptotic signaling pathways that are otherwise triggered during reperfusion injury, particularly through the Bax/Bcl-2 ratio and activation of caspase-3.

Prior studies have shown that Med1 suppression enhances Bcl-2 expression in H/R cardiomyocytes while concurrently reducing Bax expression, ultimately leading to decreased cardiomyocyte apoptosis and reduced MIRI severity [19,33]. Concurrently, Bcl-2 expression increased, while Bax expression decreased, suggesting that electroacupuncture may reduce m6A methylation in myocardial cells, contributing to its cardioprotective effects.

## 5 Conclusion

In conclusion, electroacupuncture preconditioning can enhance FTO levels in cardiomyocytes, thereby reducing m6A methylation. This reduction in m6A content is associated with decreased Med1 levels, which in turn lowers the apoptosis rate of ischemia–reperfusion-injured cardiomyocytes, ultimately mitigating the severity of MIRI. The findings suggest that electroacupuncture is a safe, effective, and straightforward approach for m6A regulation, providing new insights into MIRI pathogenesis and identifying a promising target for MIRI prevention.

## 6 Limitations

Despite these significant findings, several limitations of this study should be recognized. First, the detailed mechanisms by which electroacupuncture modulates FTO expression in cardiomyocytes require further investigation in subsequent experimental studies.

Second, although potential m6A binding sites on Med1 were identified using predictive algorithms, empirical validation of these specific loci is needed to confirm their functional relevance. Additionally, as the findings are based on murine models, translational studies are necessary to determine their applicability to human physiology. Clinical trials are essential to validate the efficacy and safety of this therapeutic approach in humans. Moreover, since EA was applied prior to ischemia in this study, which differs from clinical settings where treatment follows injury, future studies are warranted to explore its therapeutic effects when administered during or after reperfusion. Nonetheless, pre-ischemia electroacupuncture may still hold value in specific clinical scenarios where myocardial ischemia is anticipated – such as high-risk percutaneous coronary intervention involving rotablation or complex lesion preparation. In such cases, EA might serve as a prophylactic intervention to reduce procedural ischemia-induced myocardial injury. Further clinical investigation is needed to determine whether EA can mitigate risk in these planned interventions.

This study focuses on the immediate effects of EA on MIRI, such as improved EF and reduced infarct size, but its long-term efficacy and safety remain unknown. Future studies should assess the durability of these benefits and potential adverse effects. Additionally, as this study was conducted in a murine model, further research using human cardiomyocyte models or clinical trials is needed to confirm the clinical applicability of EA for MIRI.

## Abbreviations

EA: electroacupuncture
EF: ejection fraction
FTO: obesity-associated protein
IPC: ischemic preconditioning
I/R: ischemia/reperfusion
Med1: mediator complex subunit 1
MIRI: myocardial ischemia–reperfusion injury
m6A: N6-methyladenosine
ncRNAs: non-coding RNAs
PCI: percutaneous coronary intervention
PC6: Neiguan acupoints

## Supplementary Material

Supplementary Figure
