# Supplementary material for: Electroacupuncture-induced reduction of myocardial ischemia–reperfusion injury via FTO-dependent m6A methylation modulation
Source: Open Med (Wars). 2025 Aug 19;20(1):20251255. doi: 10.1515/med-2025-1255 (PMC12413781; doi:10.1515/med-2025-1255)
Supplement: Supplementary Figure [file med-2025-1255-sm.pdf]

Supplementary material

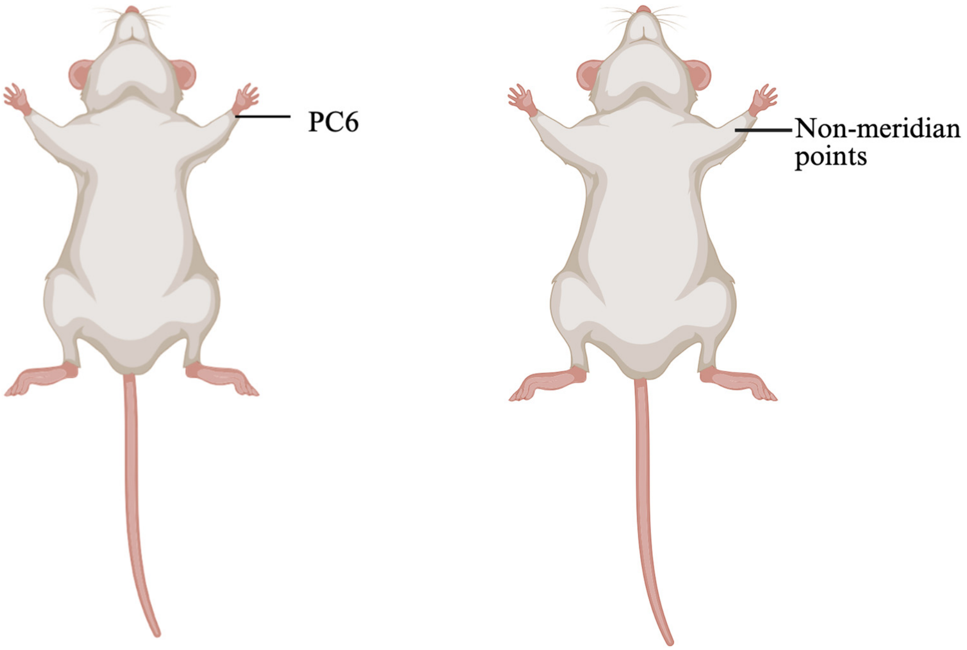

Figure S1: PC6 acupoint location.

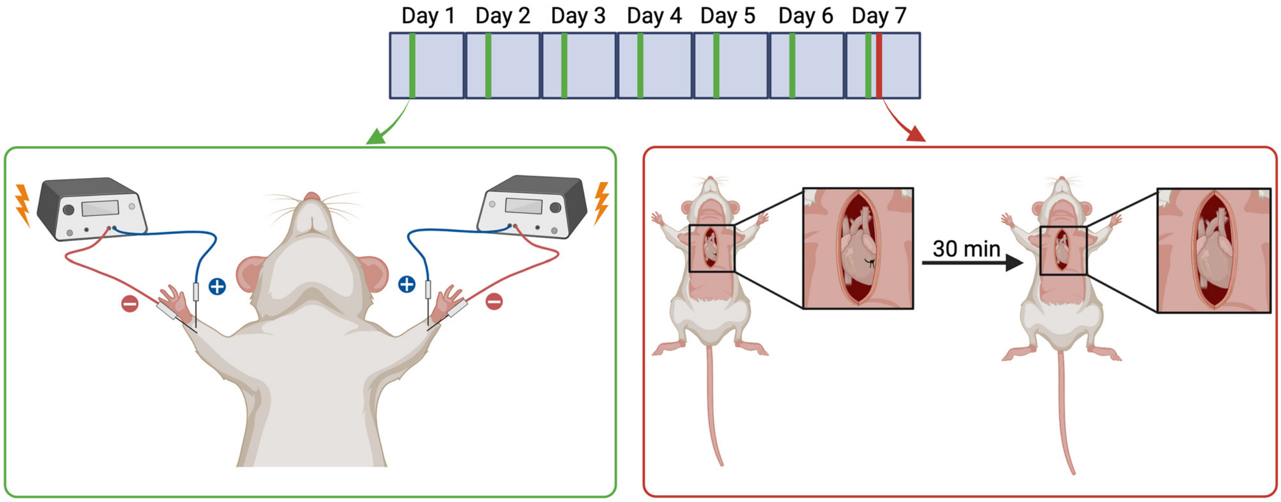

Figure S2: The timeline of electroacupuncture (EA) treatment.
